# Activation of chronic toxoplasmosis by transportation stress in a mouse model

**DOI:** 10.18632/oncotarget.13568

**Published:** 2016-11-24

**Authors:** Bang Shen, Yuan Yuan, Jianxi Cheng, Ming Pan, Ningbo Xia, Weichao Zhang, Yifan Wang, Yanqin Zhou, Junlong Zhao

**Affiliations:** ^1^ State Key Laboratory of Agricultural Microbiology, College of Veterinary Medicine, Huazhong Agricultural University, Wuhan 430070, Hubei, PR China; ^2^ Hubei Cooperative Innovation Center for Sustainable Pig Production, Wuhan 430070, Hubei, PR China

**Keywords:** Toxoplasma gondii, chronic infection, bradyzoite, reactivation, transportation stress

## Abstract

*Toxoplasma gondiiis* an obligate intracellular parasite infecting 25% of the world population and enormous number of animals. It can exist in two forms in intermediate hosts: the fast replicating tachyzoites responsible for acute infection and the slowly replicating bradyzoites responsible for life-long chronic infection. The interconversion between tachyzoites and bradyzoites plays critical roles in the transmission and pathogenesis of *T. gondii*. However, the molecular mechanisms that govern the interconversion are largely unknown. In this study, we established a chronic infection model in mice and examined the impact of transportation stress on the status of chronic infection. Our results demonstrated that, treating chronically infected mice with conditions mimicking transportation stress reduced the levels of several key cytokines that restrict the infection at chronic stage. Increased expression of the tachyzoite specific gene SAG1 (surface antigen 1) was detected in brain cysts of stress treated mice, indicating activation and conversion of bradyzoites to tachyzoites. Using this model, we identified fifteen toxoplasmic proteins that had significant abundance changes during stress induced cysts reactivation. These proteins serve as a basis for further investigation of the mechanisms governing bradyzoite conversion.

## INTRODUCTION

The apicomplexan parasite *Toxoplasma gondii* is an important zoonotic pathogen capable of infecting all warm-blooded animals and humans. It is found worldwide and estimated to infect one third of the world's population [1–3]. As a major food borne pathogen, people get infected by consuming infected undercooked meat, or by ingestion of water and vegetables that are contaminated by oocysts shed by infected cats [2, 4, 5]. Recent assessment of food borne diseases identified toxoplasmosis as the second and third leading cause of food borne illness–related deaths in U.S and Europe respectively [6]. Toxoplasmosis is listed as one of the five neglected parasitic diseases by the U.S Centers for Disease Control and Prevention (CDC), and a category B infectious disease of animals by the Ministry of Agriculture in China.

*Toxoplasma* has two forms in intermediate hosts: tachyzoites and bradyzoites. At the initial phase of infection, tachyzoites rapidly replicate to establish systemic infection in hosts. If not controlled, such infection would lead to tissue damage and inflammation responses, thus causing severe acute toxoplasmosis. Nonetheless, healthy individuals rarely develop obvious symptoms upon *Toxoplasma* infection, because of the immune clearance from the hosts [7, 8]. Under such immune pressure, some tachyzoites switch to slowly replicating bradyzoites inside host cells to avoid immune surveillance [9, 10]. Bradyzoites are commonly found in the brain and muscles, where they are enclosed by cyst wall to form tissue cysts. Once formed, tissue cysts will stay in the hosts and establish life-long chronic infection [11]. These bradyzoites containing tissue cysts are dynamic structures [12], they may rupture to release bradyzoites when the immune function of the hosts is compromised, in which case the bradyzoites can be reactivated to tachyzoites and cause acute toxoplasmosis [13, 14].

Bradyzoites have crucial roles in the transmission and pathogenesis of *Toxoplasma*, however not much is known about their biology and how they are formed or reactivated [11, 15, 16]. It is widely accepted that the status of the hosts' immune function is a critical determinant for the outcome of *T. gondii* infection [17, 18]. Stress is one important factor affecting the immune function of animals. Numerous studies have examined the effect of stress on immune functions and the outcome of diseases [19, 20]. It is evident that many pro-inflammatory cytokines such as IL-1β, IL-6, and TNF-α are involved in the modulation of neuroendocrine systems (particularly the hypothalamic-pituitary-adrenal axis) under stressful conditions. The end-effectors of the neuroendocrine systems (such as glucocorticoid hormones) also have important roles in regulating the immune functions [21–25]. As a consequence, animals under stress conditions are significantly more susceptible to many pathogens such as *Plasmodium*, *Salmonella*, *Staphylococcus*, *Pasteurella* and *Hymenolepis* [26–28].

Reduction of hosts' immune functions is thought to be able to reactivate chronic *Toxoplasma* infections and allow the differentiation of bradyzoites to tachyzoites [15, 17, 29], but the underlying mechanisms are not well understood. In this study we used a mouse model to investigate the impact of transportation stress on chronic toxoplasmosis. We established chronic infection in mice and then treated mice with conditions mimicking long distance transportation to assess their effect on chronic *Toxoplasma* infection. We also used this model, along with two-dimensional gel electrophoresis and mass spectrometry, to identify proteins that are differentially regulated during reactivation of chronic infection. The results obtained from this study provide important insights into the pathogenesis of toxoplasmosis and molecular mechanisms underlying *Toxoplasma* stage conversion.

## RESULTS

### Establishment of chronic *T. gondii* infection in mice

To establish a chronic infection model for transportation stress studies, 5-7 week old Kunming mice were infected with 10 freshly isolated cysts of the PRU strain by gavage administration. PRU is a commonly used type 2 *T. gondii* strain with intermediate virulence and efficient cyst formation ability in mice. Infected animals were monitored daily and tail blood was drawn every week to check the status of infection by a previously established ELISA method using the recombinant TgBAG1 protein [30]. The sera of mice became anti-BAG1 positive as early as 6 weeks post infection. All infected mice turned anti-BAG1 positive 10 weeks after infection (Table 1). Therefore for subsequent experiments, we used anti-BAG1 positive mice 6-8 weeks post infection as chronically infected animals.

**Table 1:** BAG1 antibody levels in the sera of infected mice determine by ELISA

Time post infection	OD_630_ of sera samples from infected mice				
	#1	#2	#3	#4	#5
2 weeks	0.122	0.205	0.144	0.194	0.131
3 weeks	0.285	0.188	0.17	0.133	0.123
4 weeks	0.129	0.194	0.302	0.126	0.146
6 weeks	**0.665**	0.184	0.121	**0.416**	**0.436**
8 weeks	**1.16**	**0.838**	0.216	**0.929**	**0.446**
10 weeks	**1.276**	**0.998**	**0.685**	**0.985**	**0.609**

Mice were infected with 10 freshly isolated cysts of the PRU strain by gavage administration, antibody levels to the BAG1 protein in the sera of infected mice were examined by ELISA. Data (means of three independent experiments) from 5 representative mice were shown and the cut-off value for positive calls is 0.38. Positive samples were indicated in bold.

### Activation of chronic *T. gondii* infection in mice undergoing transportation stress

**Figure 1:**
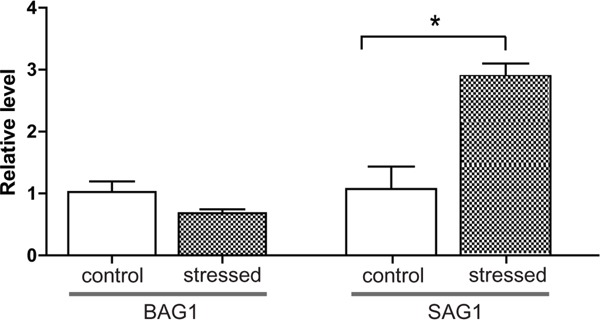
Relative expression levels of BAG1 and SAG1 in the brains of chronically infected mice before and after transportation stress The *T. gondii* PRU strain was used to infect Kunming mice, six weeks post infection, chronically infected mice were placed under conditions mimicking transportation stress for 3 days (no treatment as negative control, 3 mice each group). Subsequently, total RNA was extracted from mice brains and RT-PCR was used to determine the mRNA levels for BAG1 and SAG1. β-tubulin was used as internal control. Means ± SD from three independent experiments were graphed, *P <0.05, student's t-test.

Current knowledge predicts that reduced immune function of hosts may lead to activation of chronic *T. gondii* infection. To test this hypothesis with a transportation stress model, six chronically infected mice were evenly divided into two groups. Mice in the experimental group were placed under conditions mimicking the shaking, noisy and high temperature environment associated with long-distance transportation. To do this, the cages housing the mice were placed in a shaker that rotated at 90 r/min, the temperature was set at 35°C and mice were treated 3 hours a day for 3 days continuously. Control mice received no treatment and were housed in cages under normal conditions. After treatment, mice were sacrificed and brains (which contained *T. gondii* cysts) were isolated for RNA extraction. Total RNA was then reverse transcribed and subject to quantitative RT-PCR analysis to examine the expression levels of tachyzoite specific gene SAG1 and bradyzoite specific gene BAG1 (bradyzoite antigen 1). β-tubulin was included as internal control. Results in Figure 1 showed that, SAG1 level in the brains of infected mice increased significantly after stress treatment, indicating that some of the cysts were switching to tachyzoites.

### Cytokine production in chronically infected mice after stress treatment

**Figure 2:**
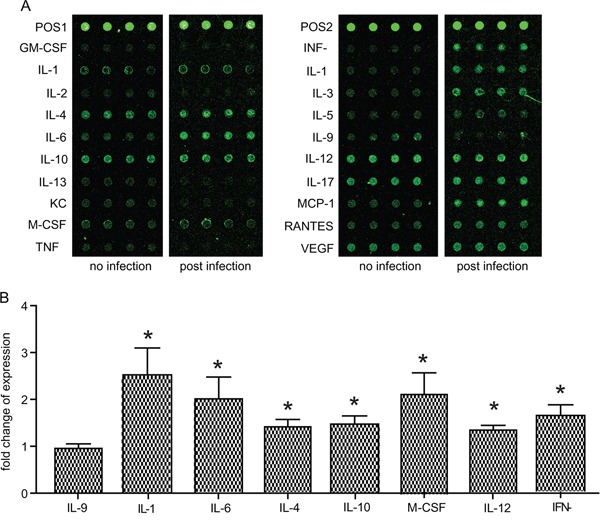
Cytokine level changes induced by chronic T. gondii infection Expression levels of the 20 indicated cytokines in peripheral blood sera of uninfected vs chronically infected mice were determined by mice cytokine array chips. **A**. Typical results from the scanning of cytokine array chips (each had four internal replicates). The data shown were from two chips, one for an uninfected mouse (no infection) and one for a chronically infected mouse (post infection). POS1 and POS2 indicated positive controls. **B**. Histogram quantification of results from three independent experiments of (A). As a way for data normalization, the average level of each cytokine in uninfected mice was set to 1. Cytokine levels in chronically infected mice after data normalization were graphed. Only the ones with significant difference (compared to uninfected control) were shown. IL-9 was included as an example with no significant changes. Means ± SD, *P <0.05, student's t-test.

Since reduced immune function is likely to be one reason for the activation of chronic *T. gondii* infection in mice undergoing transportation stress, we sought to determine the cytokine production changes in infected mice upon transportation stress. First, the cytokine profile in uninfected vs chronically infected mice was compared. Through an ELISA based mouse cytokine chip assay (Figure 2A), the levels of 20 cytokines in the peripheral blood sera were examined. Consistent with previous studies, we observed significant higher levels of IL-12 and INF-γ in infected mice, which are the major cytokines restricting acute *Toxoplasma* infection. We also detected increased levels for IL-1α, IL-4, IL-10 and M-CSF (Figure 2B), implying that these cytokines, along with IL-12 and INF-γ, play important roles in controlling the infection and confining the chronic infection in inactive state.

**Figure 3:**
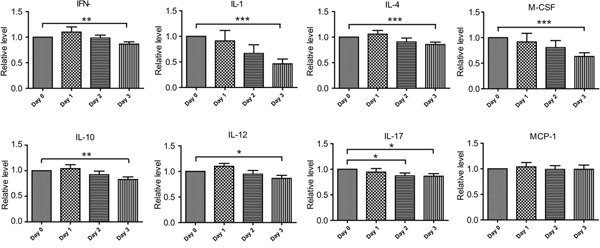
Cytokine level changes in chronically infected mice during transportation stress Kunming mice chronically infected with the PRU strain were placed under transportation stress conditions, peripheral blood was taken at day 0 (before stress), 1, 2 and 3 respectively and the levels of cytokines in the sera were determined by cytokine array chips as in Figure 2. Means ± SD from three independent experiments, *P <0.05, **P <0.005, ***P <0.0001, student's t-test.

Next, we checked the effect of stress conditions on cytokine production of chronically infected mice. To this end, chronically infected mice were placed under transportation stress for 0, 24, 48 or 72 hours, subsequently peripheral blood was taken and cytokine levels were determined by the above mentioned chip assay. Of the twenty cytokines tested, nine of them (GM-CSF, IL-5, IL-9, IL-6, IL-13, KC, TNF-α, VEGF and MCP-1) did not show any obvious changes across the stress treatment (Figure 3 and data not shown), all the rest had significantly decreased levels after three days' stress treatment (Figure 3). These results indicated that transportation stress indeed altered the immunity of infected mice, which may lead to the activation of chronic infection.

### Identification of differentially expressed *Toxoplasma* proteins during activation of chronic infection induced by host transportation stress

**Figure 4:**
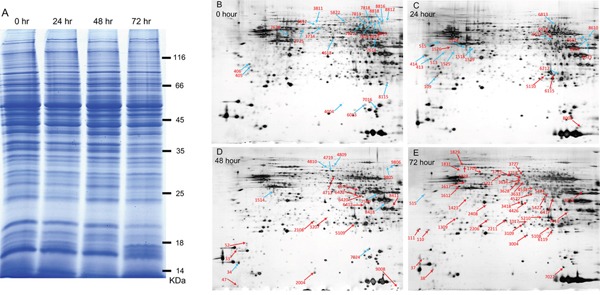
Two-dimensional gel electrophoresis to identify differentially expressed proteins during transportation stress induced activation of chronic infection Total proteins were extracted from *T. gondii* cysts (also contained host materials) of chronically infected mice underwent transportation stress for indicated amount of time and subsequently used in 2D gel electrophoresis. **A**. SDS-PAGE analysis to check the quality of protein samples extracted from brain cysts. Protein samples (20 μg each) were separated on 8% SDS-PAGE gel and stained with coomassie blue. **B-E**. Two-dimensional gel electrophoresis of protein samples in (A). A total of 150 μg proteins from each sample were first separated by isoelectric focusing electrophoresis and subsequently by SDS-PAGE, stained with sliver nitrate, scanned to determine the intensity of each spot. Proteins in the 24, 48 and 72 hour samples with abundance higher than 2 fold (up-regulated) or less than 50% (down-regulated) compared with the 0 hour sample (no stress) were identified and indicated (blue arrows indicate down-regulated proteins and red arrows indicate up-regulated ones). Experiments were repeated three times independently and only the ones with significant difference were marked on these representative gels.

The results described above suggest that transportation stress reduces the levels of key cytokines critical for controlling *T. gondii* infection, therefore leads to the activation of chronic infection. We then used this as a model to look for toxoplasmic proteins that are differentially expressed during the transition from chronic infection to acute infection. To do this, 72 chronically infected mice were divided into 4 groups (18 mice each) and placed them under transportation stress conditions for 0, 24, 48 or 72 hours. Subsequently the mice were sacrificed and cysts in the brains were purified. Roughly 10^4^ cysts were collected from each group and total proteins in these samples were extracted using the trichloroacetic acid/phenol method. A bradford method was used to measure the protein concentration of each sample and these protein samples were also examined by SDS-PAGE to ensure the quality of protein preparations (Figure 4A). Subsequently, equal amount of protein from each sample was applied to two-dimensional (2D) gel electrophoresis (one gel per sample, isoelectric focusing for the first dimension and SDS-PAGE for the second dimension). The gels were then stained with silver nitrate and scanned to get an intensity reading for each protein spot (Figure 4, 4B-4E). To identify differentially expressed proteins, the intensity of each spot on the 24, 48 and 72 hour sample gels was compared to the corresponding spot on the 0 hour sample gel. Combining the results from three independent experiments, proteins that displayed 2-fold increase or 50% decrease under stress conditions (the 24, 48 and 72 hour samples compared to the 0 hour sample) were identified as differentially expressed (Figure 4, 4B-4E). In total 109 proteins spots were identified to have significant expression changes (not all proteins are from *T. gondii*, see below), 58 of them had increased levels and 51 had decreased levels (Figure 4, 4B-4E). To determine the molecular nature of these differentially expressed proteins, the corresponding spots were cut from the gel, de-stained with K_3_Fe(CN)_6_, in-gel digested with trypsin and subsequently analyzed by MALDI-TOF/TOF mass-spectrometry. Obtained results were then imported into the MASCOT program and searched against the mouse and *Toxoplasma* genome databases to determine the corresponding proteins. From the 109 spots, we successfully identified 62 proteins with good MASCOT scores. Interestingly, the majority of these identified proteins (47 out of 62) were of mouse origin, only 15 were toxoplasmic proteins (Table 2). This is likely due to the fact that the majority of proteins in our samples were from mice brains and only a small portion was from *Toxoplasma*. These results also suggest that some of the mouse proteins were differentially regulated upon stress treatment. Of the 15 differentially expressed toxoplasmic proteins, all of them were up-regulated (compare the 72 hour sample to the 0 hour sample, Table 2). These proteins include dense granule proteins, heat shock proteins and metabolic enzymes (Table 2), indicating that there are important structural modifications and metabolic changes in parasites during activation of chronic infection.

**Table 2:** *T. gondii* proteins with significant abundance changes during stress induced activation of chronic toxoplasmosis

Spot ID ^1^	Gene	Mascot Value	Expression change ^2^		
			0 h/24 h	0 h/48 h	0 h/72 h
6420	GAPDH1	46	0.561697957	0.651378761	**0.191668879**
6422	LDH2	92	**0.463835923**	**0.480671928**	**0.318183278**
111	GRA1	203	**0.395536658**	**0.424107094**	**0.083428951**
1309	GRA7	315	**0.369398455**	0.741665978	**0.18102951**
1423	GRA9	290	0.339562473	0.451052829	**0.169774124**
1612	ACT1	356	**0.318601729**	**0.413277456**	**0.166461491**
1829	HSP70	44	0.446360187	0.497085295	**0.202121347**
1831	TGME49-078080	66	1.031312882	1.761492127	**0.471386463**
2211	BAG1	143	0.190442672	0.25667117	**0.067004656**
3004	HSP21	186	0.516842321	**0.224206999**	**0.064939766**
3109	PRX2	115	1.179950193	**0.2711159**	**0.209184601**
3727	PYK1	196	0.88264362	1.182957966	**0.284862747**
3628	ENO1	139	0.600106948	0.750584	**0.11899142**
3718	MIC13	331	0.813027221	**0.376763318**	**0.171706206**
6119	SOD	42	**0.252036272**	**0.25574946**	**0.091823386**

Protein spots on the gels shown in (Figure 4, 4B-4E) with significant abundance changes (0 h sample as the reference) were subject to MALDI-TOF/TOF mass spectrometry analysis. Subsequently the results were used for protein identification using the Mascot search engine to search the mouse and *Toxoplasma* databases. Proteins with *Toxoplasma* origin were shown.

^1^As from (Figure 4, 4B-4E).

^2^Numbers calculated from the spot intensity reads of 2D-gels in (Figure 4, 4B-4E), means of three independent experiments were presented, numbers highlighted in bold indicate significant difference (p<0.05) when compared to the 0 hour (before transportation stress) samples.

## DISCUSSION

Central to the transmission and pathogenesis of *T. gondii* is its ability to interconvert between fast replicating tachyzoites and slowly growing bradyzoites. However, the biology of bradyzoites and the molecular mechanisms governing the conversion between tachyzoites and bradyzoites are largely unknown. In this study, we established a chronic infection model in mice and tested its activation by conditions mimicking long-distance transportation. Our results indicated that stressing mice with such conditions for as short as three days led to the down regulation of a panel of cytokines, which include IL-12 and INF-γ that restrict the infection at chronic stage. Expression analysis of *Toxoplasma* genes showed that parasites isolated from mouse brain displayed increased expression level of SAG1 (Figure 1), indicating activation of chronic infection and transition to acute infection. Using this model and proteomic analysis, we also identified 15 proteins that are up-regulated during stress induced activation of chronic infection. The exact functions of these proteins during *Toxoplasma* stage conversion need to be investigated further but they form a basis to uncover the molecular mechanisms controlling bradyzoites and tachyzoites intercoversion.

Up to date, there is only limited knowledge about the formation of chronic *Toxoplasma* infection and the biology of bradyzoites [12, 31], even less is known about how bradyzoites get reactivated and switch back to tachyzoites. To address this question, we established a mouse model in which chronically infected mice were stressed with rotation, high temperature and noisy conditions that mimic long distance transportation, to check the status of chronic infection. Our results clearly showed that, when the hosts were stressed by such treatment, bradyzoites changed their gene expression patterns and started to convert to tachyzoites within three days. Using this model, we compared the proteomics of cysts isolated from mouse brains before and after stress treatment. A total of 15 *Toxoplasma* proteins were identified to have significant abundance difference upon stress treatment of the hosts. These include heat shock proteins (HSP70, BAG1 and HSP21), metabolic enzymes (LDH2, PYK1, ENO1) and so on (Table 2). Consistent with our results, heat shock proteins were previously shown to have important roles in bradyzoite transition [32]. Similarly, bradyzoites and tachyzoites were also shown to have metabolic differences, with glycolysis being the sole energy supply for bradyzoites [33]. As a consequence, most glycolytic enzymes have two or more isoforms in *T. gondii* and they are differentially regulated during stage conversion [34, 35]. In this regard, the genes we identified can serve as a basis for further investigation of the formation and reactivation of chronic infection.

Besides the complex regulation network in parasites, host environments also play important roles in determining bradyzoite conversion [16]. Reactivation of chronic toxoplasmosis to acute infection has been thought to be associated with decreased immunity of the hosts [17]. In our model, we observed that stressing the mice for as short as three days resulted in substantial changes to a panel of cytokines. These included decreased levels of IL-12 and INF-γ, the main proinflammatory cytokines that clear acute toxoplasmosis and restrict the infection at chronic stage. We also detected significant reduction of IL-1α during stress. Besides being an important proinflammatory cytokine, IL-1 is also a central regulator during stress responses to link the activities of the immune system and the neuroendocrine system [25]. Decreased level of IL-1 in mice undergoing transportation stress also suggests reduced immune functions in such animals.

The finding of stress induced reactivation of chronic infection not only allows us to investigate the mechanisms of bradyzoite conversion, but also has practical implications. With the development of animal husbandry, transporting animals from farms to farms and from farms to food processing centers is extremely common. It is also a common phenomenon that animals get sick or even die after long-distance transportation, but the reasons behind are never completely understood. Our study indicates that opportunistic pathogens such as *T. gondii* may be contributing factors to the increased morbidity and mortality after transportation. Given the high prevalence of *T. gondii* in animals, it is reasonable to predict that proper application of toxoplasmosis intervention may improve animal behavior and welfare during transportation. This possibility needs to be further tested in the future.

## MATERIALS AND METHODS

### Mice and parasites

Specific-pathogen-free (SPF), 5-7 week old female Kunming mice were purchased from the Center for Disease Control (CDC) of Hubei Province in China. They were maintained under conditions specified by the *Regulations for the Administration of Affairs Concerning Experimental Animals*. All animal work involved in this study was approved by The Scientific Ethic Committee of Huazhong Agricultural University (Approval #: HZAUMO-2016-025).

The *T. gondii* PRU strain was maintained in Kunming mice in the form of tissue cysts. To establish chronic infection in naive mice, cysts were isolated from the brains of infected mice and counted under an optical microscope, as previously described [36]. Subsequently 10 cysts were used to infect each Kunming mouse by gavage administration. From 2 weeks post infection, sera were collected from infected mice to determine their antibody levels to the bradyzoite-specific antigen BAG1. The anti-BAG1 positive mice were used as chronically infected ones for downstream experiments.

### Transportation stress simulation

Mice were placed in a temperature-constant shaker for 3 hours a day, for a total of one, two or three days. The shaker was set at 35°C and rotated at 90 r/min. Mice did not have access to food or drinking water during the three hour treatment. For the rest of the time, the mice was maintained under standard conditions.

### Determination of BAG1 and SAG1 expression levels by real time PCR

Mice were sacrificed and the brain tissues containing *Toxoplasma* cysts were removed and homogenized in liquid nitrogen. Total RNA was extracted from brain homogenates using the Trizol reagent (Life Technologies. Inc, USA). Subsequently 1 μg of total RNA was used to synthesize the first-strand cDNA, following the instructions from the SYBR Green qPCR Kit (Toyobo co., Ltd, Japan). The cDNA was then used as template for real-time PCR using the SYBR Green method, following the manufacturer's instructions. Real time PCR was performed in a 20 μl volume on the ABI ViiA 7 system (Life Technologies. Inc, USA). The primers used in real time PCR were as follow: SAG1-Fw: 5′-TGCGATGTGGCGTTATGG, SAG1-Rv: 5′-TTTTATCTGGGCAGGTGACAACT; BAG1-Fw : 5′-GACTGAGCAGTGTCCGGTTA, BAG1-Rv: TTCCGTCGGGCTTGTAATTACT; β-tubulin-Fw: 5′-CACTGGTACACGGGTGAAGGT, β-tubulin-Rv: 5′-ATTCTCCCTCTTCCTCTGCG. The cycling conditions for real time PCR were set as: 95°C for 5 min, followed by 40 cycles of 95°C for 10 sec, 60°C for 10 sec, 68°C for 10 sec. The Ct value of each sample was recorded and the 2^ΔΔCt^ method was used to assess the gene expression changes, β-tubulin was included as internal control.

### Cytokine detection

Sera obtained from peripheral blood of mice with indicated treatments were subject to a chip assay to detect the levels of 20 cytokines, which included GM-CSF, IL-1α, IL-2, IL-4, IL-6, IL-10, IL-13, KC, M-CSF, TNFα, INF-γ, IL1-β, IL-3, IL-5, IL-9, IL-12, IL-17, MCP-1, RANTES and VEGF. Samples were tested by the Mouse Cytokine Array Q1 chips from RayBiotech Company (RayBiotech, Inc. USA), following the instructions from the manufacturer. After the development of fluorescent signals in chips, the chip slides were scanned by the Axon GenePix scanner (Molecular Devices, USA) to detect the signal density for each sample. The experiment was independently repeated three times, each with four replicates. The results were graphed in Graphpad Prism 5 program (Graphpad Software, USA) and analyzed by Student's t-tests to determine the statistical differences between indicated treatments.

### Protein extraction for 2D gel elctrophoresis

Chronically infected mice were randomly divided into 4 groups (18 mice in each group) and were treated with stress conditions for 0, 24, 48 or 72 hours respectively. At the conclusion of the treatments, mice were sacrificed and brain tissues were collected for cysts enrichment. To do that, homogenized brain tissues were subject to a density gradient centrifugation in Lymphocyte Separation Medium (TBD Science, China). In a 15 ml centrifugation tube, add 5 ml lymphocyte separation medium and then add 5 ml tissue homogenates on top, the tubes were subsequently spun at 1000X g for 30min. The pellet fraction at the bottom with enriched cysts was collected for protein extraction. A trichloroacetic acid/acetone precipitation with phenol extraction method was used to isolate total proteins from enriched cysts, as previously described [37]. The concentrations of extracted proteins were measured by the Bradford protein assay before use.

### Two-dimensional (2D) gel elctrophoresis

A total of 150 μg proteins from each sample was subject to 2D gel elctrophoresis using the method described before [38]. Briefly, samples were first separated by isoelectrofocusing on the pH 3-10 NLIPG (non-linear immobilized pH gradient) strips (GE healthcare, Sweden), with the following conditions: 50V for 12 hours, followed by 500 V for 1 hour, followed by 1000 V for 1 hour, followed by 1000∼10000 V for 1 hour, and finally10000 V for 11 hours. The IPG strips were then incubated in 2D equilibration buffer (6M Urea; 30%Glycerol; 2%SDS; 0.05M Tris-HCl, pH 6.8) containing 1% dithiothretiol (freshly added before use) for 15 min with gentle shaking, followed by a second incubation with 2D equilibration buffer containing 2.5% iodoacetamide (freshly added before use) for 15 min. Then the strips were rinsed in SDS-gel running buffer before the second dimentional elctrophoresis on 12% SDS-gels, which was performed under 100 V for 45 min, followed by 200 V until completion. Protein gels were subsequently stained by silver staining. Each sample was prepared and analyzed three times independently.

### Analysis of 2D gel results

Silver stained gels were scanned by ImageScanner (GE healthcare, Sweden) to obtain images with a resolution of 300 dpi (dot/inch). Images were then analyzed by the PDquest 8.0 software to examine the density of each protein spot. Density readings for each protein spot in three independent experiments were combined and used in statistical analysis to check the abundance difference under different treatments (the 0 hour samples were used as a reference). Any spot with a 2 fold density difference (compared to 0 hour) and a P value less than 0.05 (Student's t test) was considered to have significant abundance difference. Such protein spots were picked from the gels and identified by mass spectrometry.

### Protein identification by MALDI-TOF/TOF

The protein spots cut from the gels were destained with 30 mM K_3_Fe(CN)_6_ and 100 mM Na_2_S_2_O_3_ (vol/vol=1:1) and in-gel digested with trypsin (Promega, USA) for 20 hours at 37°C. The peptide mix was then extracted with 60% Acetonitrile/0.1% trifluoroacetic and vacuum dried. The dried material was dissolved in 20% acetonitrile for mass spectrometry analysis using the 4800 Plus MALDI-TOF/TOF Analyzer (Life Technologies. Inc, USA) equipped with a ND:YAG laser source. Peptide mass fingerprint obtained from mass spectrometry analysis was used for protein identification using the Mascot search engine to search the databases of National Center for Biotechnology Information non-redundant (NCBI-nr) and ToxoDB (www.toxodb.org).

## Abbreviations

INF-γ: interferon gamma
IL: interleukin
KC: keratinocyte chemoattractant
TNF-α: tumor necrosis factor alpha
VEGF: vascular endothelial growth factor
MCP-1: monocyte chemoattractant protein-1
M-CSF: macrophage colony-stimulating factor
RANTES: regulated on activation, normal T cell expressed and secreted
BAG1: bradyzoite antigen 1
SAG1: surface antigen 1
MALDI-TOF/TOF: matrix assisted laser desorption ionization-time of flight.
